# A phase I study using bortezomib (Velcade), cladribine, and rituximab in treating patients over 50 years old with mantle cell lymphoma

**DOI:** 10.3389/fonc.2024.1449401

**Published:** 2024-12-16

**Authors:** Jeffrey J. Pu, Kristin N. Berger, Chunlei Zheng, Nhan Do, David F. Claxton, W. Christopher Ehmann, Joseph J. Drabick, Haiquan Li, Thomas P. Loughran, Elliot M. Epner

**Affiliations:** ^1^ Division of Medicine, VA Boston Healthcare System, Boston, MA, United States; ^2^ Brigham & Women’s Hospital, Harvard Medical School, Boston, MA, United States; ^3^ New York Presbyterian Hospital, Weill Cornell Medicine, New York, NY, United States; ^4^ Department of Medicine, Boston University School of Medicine, Boston, MA, United States; ^5^ Penn State Hershey Cancer Institute, Pennsylvania State University College of Medicine, Hershey, PA, United States; ^6^ Department of Biosystems Engineering, University of Arizona, Tucson, AZ, United States; ^7^ Department of Medicine, University of Virginia National Cancer Institute (NCI) Designated Comprehensive Cancer Center, Charlottesville, VA, United States

**Keywords:** mantle cell lymphoma (MCL), epigenetic, combination (combined) therapy, phase I trial, cladribine

## Abstract

Cladribine indirectly downregulates methylation of DNA, RNA, and histones by blocking the transfer of methyl groups from *S*-adenosyl-methionine. The cladribine and rituximab combination showed a synergetic effect in treating B-cell lymphomas. Bortezomib (Velcade) is a Food and Drug Administration (FDA)-approved proteasome inhibitor for treating mantle cell lymphoma (MCL). In this single-arm, phase I study, the safety, dose-limiting toxicity, and clinical activity of bortezomib, cladribine, and rituximab (VCR) combination treatment were evaluated in elderly MCL patients. Potential DNA methylation biomarkers for VCR treatment were also proposed. A standard 3 + 3 dose-escalation scheme was designed to determine the maximum tolerated dose of cladribine. The therapy consisted of six 28-day cycles. Most patients tolerated this regimen well. The overall response (OR) rate was 84.6%, and the complete remission (CR) rate was 84.6%. In the newly diagnosed subject cohort, the OR and CR were 100%, the 2-year overall survival rate was 84.6%, and the progression-free survival rate was 76.9%. The median age was 64 (54–81). The median time to first response was 3 (2.1–7.4) months. The median follow-up time was 43 (9–60) months. Low-grade hematological toxicity and mild fatigue were observed. No severe systemic toxicity was observed. Five hypermethylated regions located at gene promoters were identified as potential biomarkers for an effective treatment response. In conclusion, the VCR combination is a well-tolerated, low-toxicity, and highly effective regimen for the elderly with untreated MCL.

**Clinical Trial Registration:**

ClinicalTrials.gov, identifier NCT01439750.

## Introduction

Mantle cell lymphoma (MCL) is a malignancy of monomorphically small to medium-sized B lymphocytes. The majority of MCL cells are CD5+ and CD23− and exhibit the t(11;14) chromosomal translocation leading to deregulated expression of cyclin D1 (1, 2). MCL is considered incurable with current available chemotherapies. Median survival estimates are 3 to 7 years, with even shorter survival times in patients with blastoid variant type disease and with higher lymphoma cell proliferation rates (3–5).

In the last decade, a multitude of innovative therapeutic strategies that employ epigenetic modulation, signal transduction pathway intervention, or immunotherapeutic suppression have been emerging in managing MCL (1, 2). Although there is no consensus on first-line treatment, chemotherapy is still considered the backbone of first-line treatment. Several chemotherapy regimens have been used for MCL management, including aggressive regimens such as high-dose cyclophosphamide, vincristine, doxorubicin, dexamethasone plus rituximab (R-hyper-CVAD), as well as cyclophosphamide, doxorubicin, vincristine, and prednisone in combination with rituximab (R-CHOP). In a study by Romaguera et al. (6), which employed R-hyper-CVAD, the complete remission (CR) rate was found to be 87% after the completion of six cycles of chemotherapy, with a failure-free survival (FFS) rate of 64% and overall survival (OS) of 82% at 3 years. Despite generating high overall response (OR) rates, these regimens are not curative. In addition, the regimens are highly toxic. Of the 97 patients in the Romaguera trial, there were eight toxicity-related deaths (8.3%), as well as four cases of treatment-related acute leukemia, three of which were fatal. As a result, many patients with MCL are not eligible to undergo treatments with these aggressive regimens due to either advanced age or comorbidities.

In an effort to find less toxic treatment regimens, the North Central Cancer Treatment Group used the combination of rituximab and cladribine in previously untreated MCL patients (7). The study resulted in an OR rate of 66% and a CR rate of 52%. Only three of 15 patients who achieved CR developed recurrent disease at a median follow-up time of 21.5 months. The high response rates and long response duration of this well-tolerated regimen were promising, which raises the question of whether responses could be improved by the inclusion of an additional agent targeting an MCL-related signal pathway.

Bortezomib (Velcade), a small-molecule proteasome inhibitor, is currently approved by the Food and Drug Administration (FDA) for the treatment of MCL (8, 9). Literature demonstrated that adding bortezomib to standard immunochemotherapy regimens could benefit those newly diagnosed MCL patients (10). Most recently, data from our laboratory and other laboratories showed that hypomethylating agents synergistically increase the treatment efficacy (11–14). We herein present the data of a phase I study conducted to evaluate the safety and efficacy of bortezomib, cladribine, and rituximab (VCR) combination regimen in treating MCL, especially for those newly diagnosed elderly patients, as the first-line therapy. We also evaluated the dose-limiting toxicity (DLT) of cladribine, and we identified the maximum tolerated dose (MTD).

## Methods

### Patient eligibility

Patients with MCL who met the following criteria were eligible for participation in this study: diagnosed as MCL with bone marrow involvement, either treatment naïve, relapsed, or refractory MCL; had received no treatment in the 14 days prior to study entry; had an Eastern Cooperative Oncology Group (ECOG) performance status score of 3 or less; had a platelet count of at least 50 × 10^9^ cells/L within 14 days before enrollment if not related to disease; had an absolute neutrophil count of at least 1 × 10^9^ cells/L within 14 days before enrollment if not related to disease; had a calculated or measured creatinine clearance of >35 mL/minute within 14 days before enrollment; had less than grade 2 peripheral neuropathy within 14 days before enrollment; and had less than 1.5 times the upper limit of normal bilirubin. This study was approved by the Institutional Review Board (IRB) and was compliant with institutional guidelines and the Declaration of Helsinki. Informed written consent was obtained prior to patient enrollment.

### Study design

**Figure 1:**
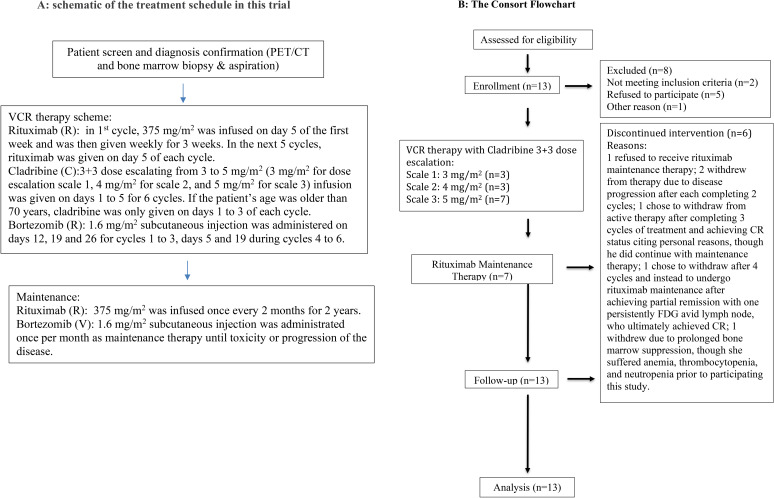
**(A)** Schematic of the treatment schedule in this trial. **(B)** The CONSORT flowchart.

A single-arm, open-label, investigator-initiated phase 1 clinical trial was performed to assess the safety and efficacy of combination treatment using bortezomib, cladribine, and rituximab in MCL patients (**Figure 1**). This study employed a standard 3 + 3 dose-escalation scheme designed to determine the MTD of cladribine within this regimen. The therapy consisted of six cycles, with 28 days in each cycle. During the first cycle, rituximab 375 mg/m^2^ infusion was administered on day 5 of the first week and was then given weekly for 3 weeks. In the next five cycles, rituximab was given on day 5 of each cycle and then once every 2 months as maintenance therapy. Cladribine 3–5 mg/m^2^ (3 mg/m^2^ for dose-escalation scale 1, 4 mg/m^2^ for scale 2, and 5 mg/m^2^ for scale 3) infusion was given on days 1 to 5 for six cycles. If the patient’s age was older than 70 years, cladribine was only given on days 1 to 3 of each cycle. Bortezomib 1.6 mg/m^2^ subcutaneous injection was administered on days 12, 19, and 26 during cycles 1 to 3, days 5 and 19 during cycles 4 to 6, and then once per month as maintenance therapy until toxicity or progression of the disease. Dosing and duration of growth factor support with filgrastim or pegfilgrastim were determined by the treating physician for neutropenia.

The primary endpoint of this study was to investigate the DLT and safety of this regimen in patients with MCL. The secondary endpoints included OR and CR rates, response duration, and progression-free survival (PFS) and OS rates. PET/CT evaluation of lymphadenopathy status was mainly used as the response assessing method. The first PET/CT evaluation was performed within 4 weeks of initiating treatment; the second PET/CT evaluation was performed after completing the first two or three cycles of chemotherapy. Then, PET/CT evaluation was performed every 6 months for 2 years and once a year thereafter if the patient’s lymphoma was still in stable or remission status. The Deauville score was used for interpreting PET results (15). The Lugano criteria were used for post-treatment response assessments (16). Lugano 5-point scale was used to define the response assessment: a score of 1 or 2 indicates a complete metabolic response (CR), a score of 3 or 4 shows a partial metabolic response (PR), and a score of 5 demonstrates progressive metabolic disease (PD). This study is registered with ClinicalTrials.gov (NCT01439750).

### DNA methylation analysis

The bone marrow aspiration samples were collected each time the patient underwent bone marrow biopsy and aspiration. The mononuclear cells were then extracted for study. Following the completion of the clinical trial, a DNA methylation assay was performed on four patients to assess the biological factors involved in treatment response. All these four patients presented with extensive MCL bone marrow involvement before receiving treatment. Two patients achieved CR after treatment (responders), and the other two did not respond to the treatment with disease progression while on treatment (non-responders). The samples JP01, JP02, JP07, and JP08 were collected from two responders: JP01 and JP07 right before starting treatments and JP02 and JP08 at post-treatments. The samples JP03, JP04, JP05, and JP06 were collected from two non-responders: JP03 and JP05 right before starting treatments and JP04 and JP06 post-treatment. Reduced representation bisulfite sequencing (RRBS) (17) assay was used to measure DNA methylation at CpG sites. In brief, the extracted DNA was treated with restriction enzymes to generate sequence-specific fragments, which were further selected for bisulfite conversion and sequencing. Bismark (18) was used for genomic mapping and methylation calling, and a total of ~4 million methylation sites were obtained.

All DNA methylation analyses were performed using R (version 4.2.1) (19). Principal component analysis (PCA) was used to obtain overall methylation profiles for patients. There are several analytic tools available for differentially methylated region (DMR) analysis. The BSseq package was used in this study since it can deal with the small sample size and take biological variability into account (20). The small sample size also caused difficulty in statistically identifying genomic regions with small differences. To increase the statistical power and reliability, only promoter regions that were identified using GenomicFeatures (21) were considered in our analysis. DMR was defined as a region that has at least two measured loci, and the mean difference in methylation level between two groups is higher than 0.3. Quantile cutoff (lower, 0.025; upper, 0.075) of t-statistic was used for DMR identification. These DMRs were further mapped to specific genes using Ensembl in biomaRt (22, 23).

### Statistical methods

All patients enrolled in this study were assessed to determine OR and CR rates, response duration, and PFS and OS rates. The duration of response to treatment was measured from the date of the first observed remission to the time at which relapse was noted. PFS was measured between the time of first treatment and the time of disease progression, relapse, or death. OS was measured from the time of first treatment to the time of death or last known survival date. Patients were censored at the date they were last known to be alive in the definition of OS. The Kaplan–Meier method was used to estimate times to events (in months).

## Results

### Patient characteristics

Thirteen patients were enrolled in this 24-week dose-escalation study (**Table 1**). Most patients were male (11/13), with a median age of 64 years (range 54–81 years). Of 13 patients, nine (69%) had low-risk Mantle Cell Lymphoma International Prognostic Index (MIPI) scores, and four (31%) had intermediate-risk MIPI scores. In terms of individual MIPI risk factors, eight (62%) patients were >60 years old, all patients were identified as having ECOG performance status from 0 to 3, 13 (100%) patients had elevated levels of lactate dehydrogenase, and eight (62%) patients had elevated white blood cells counts.

**Table 1:** Patient baseline characteristics.

Characteristic	N = 13
Median age (range), years	64 (54–81)
Sex, male/female	11/2
ECOG performance score, n (%)	
0	1 (7.7)
1	6 (46.2)
2	4 (30.8)
3	2 (15.3)
MIPI score, n (%)	
Low (<5.7)	9 (69)
Intermediate (5.7–6.2)	4 (31)
High (>6.2)	0 (0)
Risk factors, n (%)	
Age >60 years	8 (62)
ECOG ≥ 2	6 (46)
LDH/ULN > 6.7	13 (100)
WBC > 6.7	8 (62)
MCL classifications	
Classic	11 (85)
Aggressive/blastoid variant	2 (15)

ECOG, Eastern Cooperative Oncology Group; MIPI, Mantle Cell Lymphoma International Prognostic Index; LDH, lactate dehydrogenase; ULN, upper limits of normal; WBC, white blood cell; MCL, mantle cell lymphoma.

Most patients (10/13) never received prior treatment for MCL. Of the relapsed MCL patients who previously received treatment, two patients were experiencing their first relapse following previous treatment with rituximab and bendamustine. The third patient experienced a third relapse following previous treatment with R-CHOP, RDHAP, hyper-CVAD, and allogeneic stem cell transplantation (SCT) therapy.

The dose-escalation study tested cladribine in doses ranging from 3 to 5 mg/m^2^ on days 1 through 5 of six 28-day cycles. Of the 13 patients, three received 3 mg/m^2^ of cladribine on dose scale 1, three received 4 mg/m^2^ of cladribine on dose scale 2, and seven received 5 mg/m^2^ of cladribine on dose scale 3. Of the 13 patients, seven completed the 24-week dose-escalation study followed by rituximab maintenance therapy as described in the Study Design section. One patient refused to receive rituximab maintenance therapy. Two patients withdrew from therapy due to disease progression after each completing two cycles. One chose to withdraw from active therapy after completing three cycles of treatment and achieving CR status, citing personal reasons, although he did continue maintenance therapy. Another one also chose to withdraw after four cycles and instead underwent rituximab maintenance after achieving partial remission with one having persistently (18) F-fluorodeoxyglucose (FDG)-avid lymph node, who ultimately achieved CR. One patient withdrew due to prolonged bone marrow suppression, although she suffered anemia, thrombocytopenia, and neutropenia prior to participating in this study.

### Safety

No subject experienced DLT while receiving cladribine dose scales 1 or 2. One subject who was receiving cladribine on dose scale 3 suffered infectious colitis during the second cycle of therapy and was labeled as possibly experiencing DLT. The patient died 7 months later as a result of multiple organ failure after experiencing disease progression. Additional patients were recruited to the cladribine dose scale 3 cohort, and no one experienced DLT.

**Table 2** summarizes the most frequently reported adverse events (AEs) with incidence rates in each grade. All AEs were grade 1 or 2 in severity other than single patient reports of cellulitis, diarrhea, neuropathy, fatigue, and hyperglycemia. Neuropathy in this study is defined as having burning, numbness, tingling, or painful sensations in the arms, hands, legs, or feet. The most common non-hematological AEs of any grade were neuropathy (54%), fatigue (54%), diarrhea (39%), constipation (38%), and hyponatremia (38%). No patient experienced tumor lysis syndrome. Only one patient discontinued the treatment due to prolonged bone marrow suppression caused by pancytopenia as previously mentioned. Of the five patients who discontinued treatment prior to completion of all six cycles, the median number of treatment cycles prior to discontinuation was 2 (range 2–4).

**Table 2:** Common adverse events (n = 13) on patients participating in the trial.

	Grade 1 (%)	Grade 2 (%)	Grade 3 (%)	Grade 4 (%)
Hematological events				
Anemia	4 (31)	3 (23)	0 (0)	0 (0)
Neutropenia	5 (0)	6 (15)	0 (0)	0 (0)
Febrile neutropenia	1 (8)	0 (0)	0 (0)	0 (0)
Thrombocytopenia	3 (23)	1 (8)	0 (0)	0 (0)
Leukopenia	3 (23)	5 (38)	0 (0)	0 (0)
Lymphopenia	2 (15)	10 (77)	0 (0)	0 (0)
Non-hematological events				
Fever	1 (8)	1 (8)	0 (0)	0 (0)
Chills	3 (23)	0 (0)	0 (0)	0 (0)
GFR decrease	1 (8)	0 (0)	0 (0)	0 (0)
Cellulitis	0 (0)	0 (0)	1 (8)	0 (0)
Diarrhea	4 (31)	1 (8)	0 (0)	0 (0)
Dyspnea on exertion	3 (23)	0 (0)	0 (0)	0 (0)
Hyperuricemia	2 (15)	0 (0)	0 (0)	0 (0)
Increased alkaline phosphatase	1 (8)	0 (0)	0 (0)	0 (0)
Increased ALT	1 (8)	1 (8)	0 (0)	0 (0)
Increased AST	4 (31)	0 (0)	0 (0)	0 (0)
Increased total bilirubin	2 (15)	0 (0)	0 (0)	0 (0)
Increased LDH	1 (8)	0 (0)	0 (0)	0 (0)
Hives	1 (8)	0 (0)	0 (0)	0 (0)
Watery eyes	1 (8)	0 (0)	0 (0)	0 (0)
Bone pain	0 (0)	1 (8)	0 (0)	0 (0)
Neuropathy	2 (15)	4 (31)	1 (8)	0 (0)
Myalgia	3 (23)	0 (0)	0 (0)	0 (0)
Fatigue	3 (23)	3 (23)	1 (8)	0 (0)
Stye	0 (0)	1 (8)	0 (0)	0 (0)
Loss of balance	1 (8)	0 (0)	0 (0)	0 (0)
Joint/back pain	0 (0)	3 (23)	0 (0)	0 (0)
Bradycardia	1 (8)	0 (0)	0 (0)	0 (0)
Constipation	3 (23)	2 (15)	0 (0)	0 (0)
Upper respiratory infection	0 (0)	1 (8)	0 (0)	0 (0)
Weight loss	0 (0)	1 (8)	0 (0)	0 (0)
Abdominal pain	2 (15)	1 (8)	0 (0)	0 (0)
Tonsillitis	0 (0)	1 (8)	0 (0)	0 (0)
Hypocalcemia	2 (15)	0 (0)	0 (0)	0 (0)
Nausea	4 (31)	0 (0)	0 (0)	0 (0)
Mouth sores	1 (8)	0 (0)	0 (0)	0 (0)
Cold intolerance	1 (8)	0 (0)	0 (0)	0 (0)
Insomnia	1 (8)	1 (8)	0 (0)	0 (0)
Loss of appetite	1 (8)	1 (8)	0 (0)	0 (0)
Vomiting	3 (23)	0 (0)	0 (0)	0 (0)
Lower extremity edema	1 (8)	1 (8)	0 (0)	0 (0)
Hypoalbuminemia	1 (8)	0 (0)	0 (0)	0 (0)
Hypomagnesemia	1 (8)	0 (0)	0 (0)	0 (0)
Hyponatremia	5 (38)	0 (0)	0 (0)	0 (0)
Hyperglycemia	2 (15)	1 (8)	1 (8)	0 (0)
Hypernatremia	1 (8)	0 (0)	0 (0)	0 (0)
Weakness	1 (8)	3 (23)	0 (0)	0 (0)
Lightheadedness	1 (8)	0 (0)	0 (0)	0 (0)
Rash	2 (15)	0 (0)	0 (0)	0 (0)
Avascular necrosis	0 (0)	1 (8)	0 (0)	0 (0)

Data are number of patients (%).

GFR, growth factor receptor; ALT, alanine aminotransferase; AST, aspartate aminotransferase; LDH, lactate dehydrogenase.

### Efficacy

**Figure 2:**
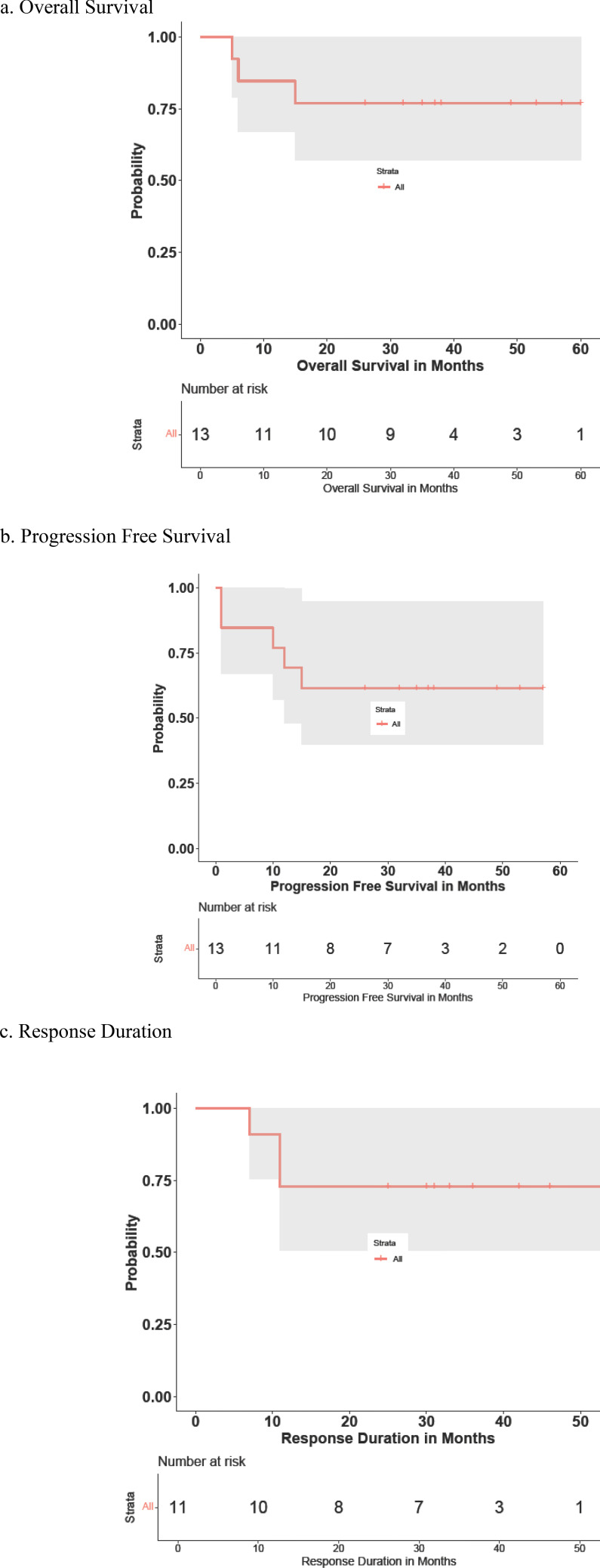
Patients’ **(A)** overall survival (OS), **(B)** progression-free survival (PFS), and **(C)** response duration (RD). OS or PFS months count from the treatment starting date to the event happening date, which were death for OS curve, and relapse, progression, or death for PFS curve. RD counts similarly to PFS curve, except the starting point is from the first response date when remission happened.

**Table 3** summarizes each cohort’s best response to therapy. Of the 13 patients in this study, the OR rate was 11/13 patients (84.6%, 95% confidence interval 57.8–95.7). CR was reported in 11/13 patients (84.6%, 95% confidence interval 57.8–95.7). In the newly diagnosed subject cohort, the ORR and CR rates were both 100% (10/10). The median time to first response was 3 (2.1–7.4) months. The median follow-up time was 43 (9–60) months. The 2-year and 4-year OS rates in this cohort were 85% (11/13) and 66.7% (4/6), respectively (**Figure 2A**). The 1-year, 2-year, and 4-year PFS rates in this same cohort were 77% (10/13), 60% (9/13), and 50% (3/6), respectively (**Figure 2B**). The median duration of response, PFS, and OS had not been reached at the time of the current report. Two patients died during the period of the study due to disease progression.

**Table 3:** Response rates in newly diagnosed and relapsed MCL patients receiving bortezomib, cladribine, and rituximab.

	Receiving 3 mg/m^2^ (n = 3)	Receiving 4 mg/m^2^ (n = 3)	Receiving 5 mg/m^2^ (n = 7)	Newly diagnosed (n = 10)	Overall (n = 13)
Patients with relapsed MCL	1 (33)	0 (0)	2 (29)	0 (0)	3 (23)
ORR	3 (100)	3 (100)	4 (57)	9 (90)	11 (84.6)
Best Response					
CR	3 (100)	3 (100)	5 (71)	10 (100)	11 (84.6)
PR	0 (0)	0 (0)	0 (0)	0 (0)	0 (0)
DP	0 (0)	0 (0)	2 (29)	0 (0)	2 (15)
Median time to first response (range, months)	2.5 (2.1–3.2)	6.8 (2.2–7.4)	3.0 (2.4–6.8)	3.1 (2.1–7.4)	3.0 (2.1–7.4)
1-year PFS	2 (67)	3 (100)	5 (71)	9 (90)	10 (77)
2-year PFS	1 (33)	3 (100)	4 (57)	8 (80)	9 (69)
1-year OS	3 (100)	3 (100)	5 (71)	10 (100)	11 (84.6)
2-year OS	3 (100)	3 (100)	4 (57)	9 (90)	11 (84.6)

Dose cohorts received 3–5 mg/m^2^ of cladribine. Data are number of patients (%).

PR, partial remission; DP, disease progression; MCL, mantle cell lymphoma; ORR, overall response rate; CR, complete response; PFS, progression-free survival; OS, overall survival.

Four patients experienced disease progression, including two deceased patients with relapsed disease who did not respond to treatment and two patients who experienced relapse following complete remissions (**Figure 2B**). One patient’s death was not preceded by disease progression. Of the two patients who experienced disease progression without achieving remission, both suffered progression within 2 months of beginning this treatment, and both had the blastoid variant type of MCL and suffered from relapsed disease at the time that they were enrolled in this study.

Of the patients who achieved a response, only two experienced disease relapses, with response durations of 7.6 and 11.2 months. The remaining patients on the response duration curve (**Figure 2C**) were censored, with one patient’s remission time currently lasting longer than 50 months after first reaching CR.

### Biological differences

**Figure 3:**
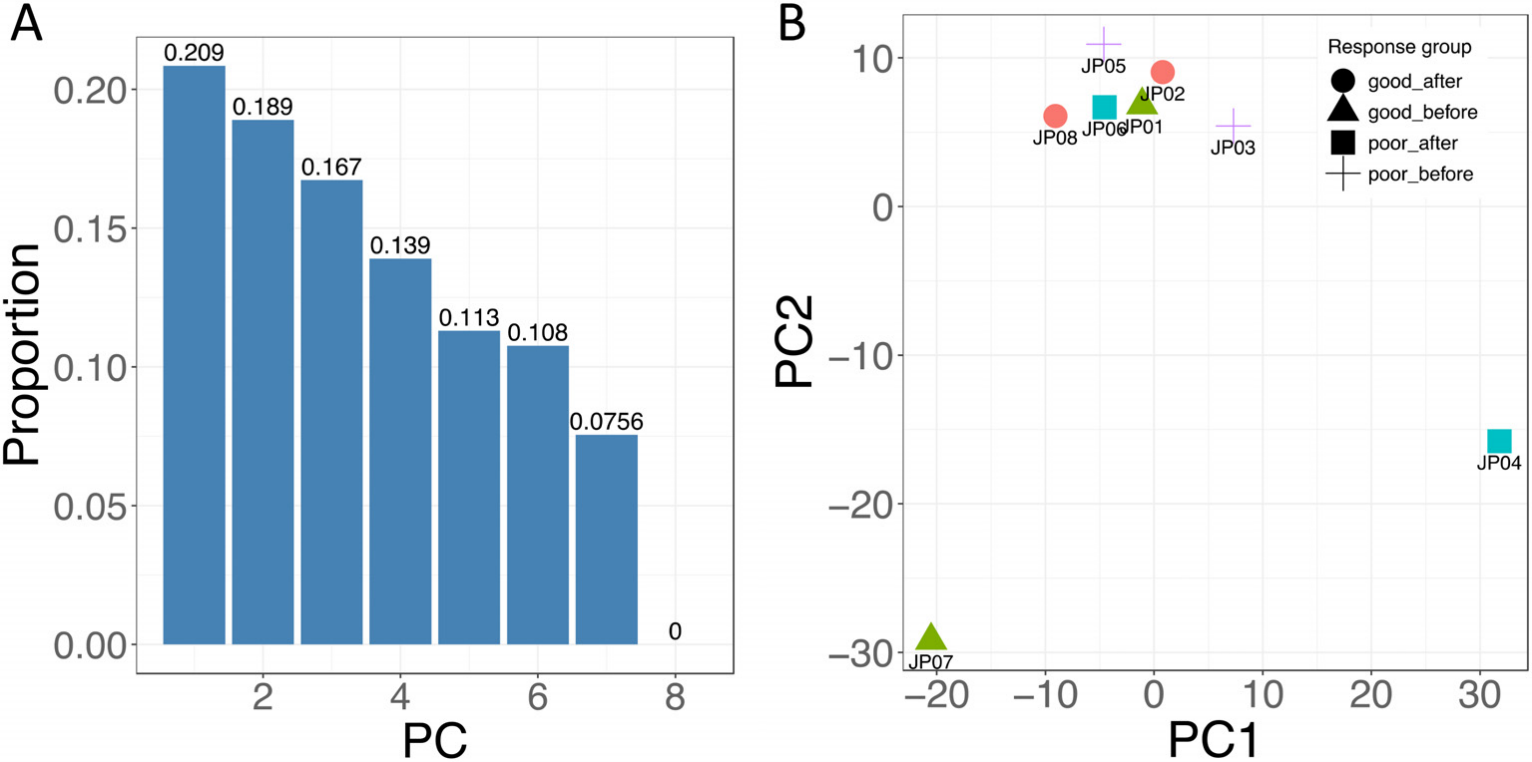
Principal component analysis (PCA) of DNA methylation. **(A)** The proportion of variance plot displays the percentage of variance explained by the eight principal components. **(B)** The PCA plot illustrates the first and second components for each sample, with samples grouped by response status and treatment time points, represented by different shapes. Samples JP01 and JP02 were from one responder, and JP07 and JP08 were collected from another responder; JP01 and JP07 were collected just before treatment (good-before), and JP02 and JP08 were collected after treatment (good-after). Samples JP03 and JP04 were from one non-responder, and JP05 and JP06 were from another non-responder, with JP03 and JP05 collected before treatment (poor-before) and JP04 and JP06 collected after treatment (poor-after). For instance, JP01 and JP07 are represented as triangles, indicating that they are from responders prior to treatment.

An initial attempt was made to determine whether there were any overall differences in methylation values between the subjects using PCA. **Figure 3A** shows that the high-dimension data can be represented by the first seven components. Although the first two components only explained approximately 40% of the variance of our data, which prevented us from visualizing them in a two-dimensional space efficiently (**Figure 3B**), we still observed the treatment-induced methylation changes. Prior to treatment, a sample from a responder (JP07) showed differences compared to the other three patients. After the treatment, the methylation from this patient had a big change (JP08). We also observed that the methylation from one non-responder had a large change in the opposite direction after treatment (JP04 vs. JP03).

**Figure 4:**
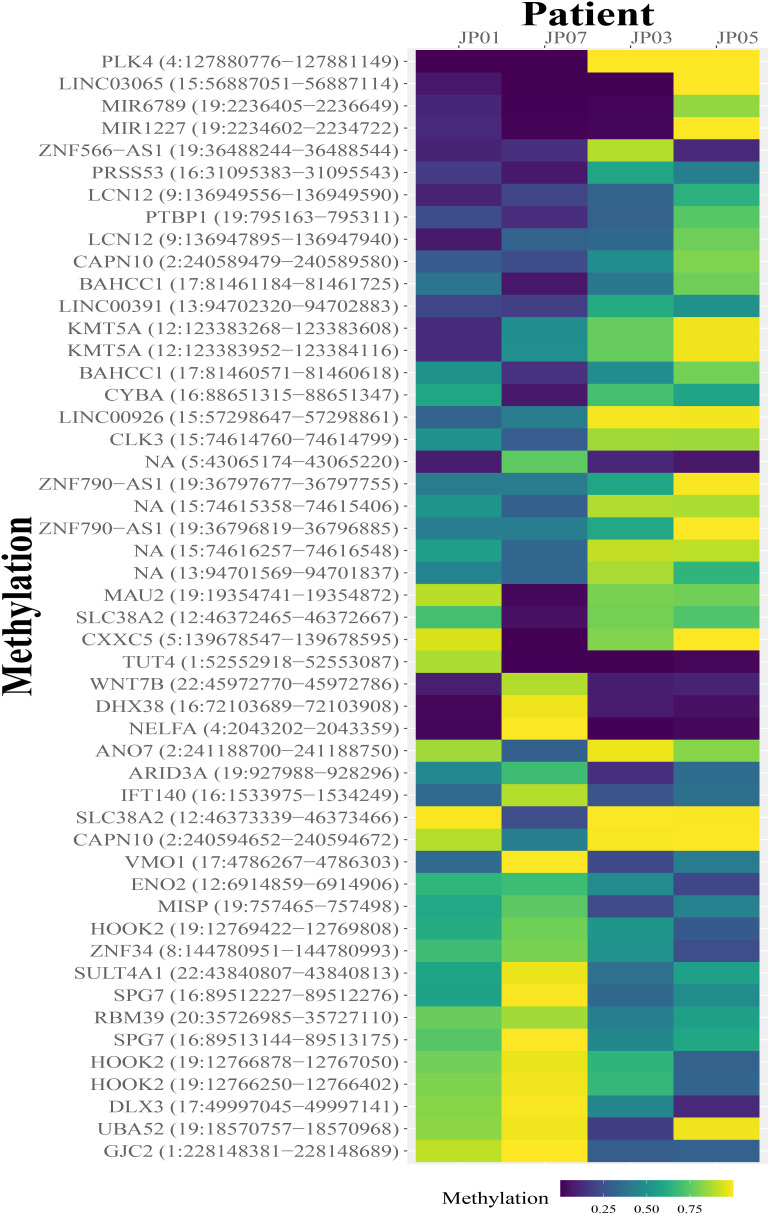
Heatmap shows differentially methylated regions (DMRs) between samples from good responders (JP01 and JP07) and poor responders (JP03 and JP05). Each DMR is labeled with its genomic location and mapped to individual genes. Several DMRs are unable to be mapped to genes.

DMR analysis was then performed between responders and non-responders before treatment, and a total of 50 DMRs within gene promoter regions were identified, including both hypermethylated and hypomethylated regions (**Figure 4**).

**Figure 5:**
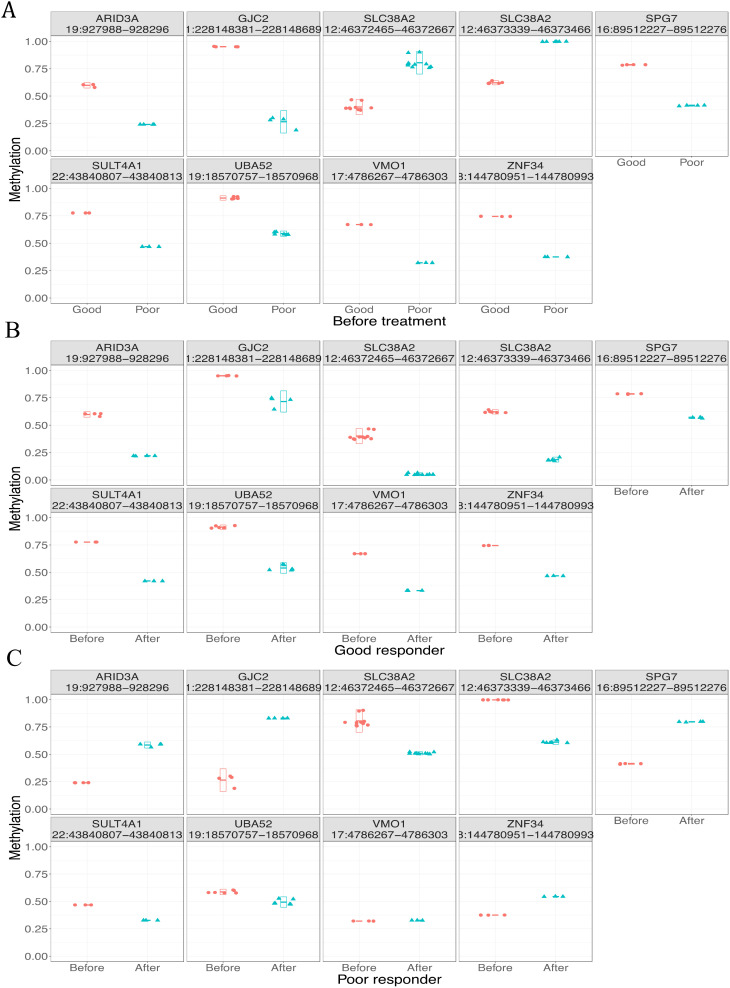
Methylation profiles of candidate differentially methylated regions (DMRs) in different groups. **(A)** Good and poor responders before treatment. **(B)** Before and after treatment in good responders. **(C)** Before and after treatment in poor responders.

In this study, the methylation biomarkers for treatment response should meet two criteria: 1) they are differentially methylated between responders and non-responders before treatment; 2) they respond to treatment differently between responders and non-responders after treatment. We identified nine candidate DMRs (**Figure 5A**). All these regions were hypermethylated before treatment and hypomethylated after treatment in responders (**Figure 5B**). When we examined the methylation profiles of non-responders, five of nine DMRs showed hypomethylated status before treatment while increased methylation or no change after treatment (subpanels 1, 2, 5, 8, and 9 in **Figure 5C**); the remaining four DMRs showed the opposite trend (subpanels 3, 4, 6, and 7 in **Figure 5C**). Thus, the five DMRs differentially responded to treatment between responders and non-responders, indicating that they are potential methylation biomarkers for treatment response.

## Discussion

The current most utilized regimens to initiate MCL treatment, including R-CHOP and R-hyper-CVAD, produce high overall response rates, although they often result in late relapse and are extremely toxic (6, 24–26). Due to the toxicity of these regimens, it is challenging to treat elderly patients with MCL, particularly those with comorbidities.

Prior trials have demonstrated the capabilities of both rituximab and cladribine as monotherapies in treatment (27–29). In addition to patients treated in the study by Inwards et al. (7) (2008), rituximab and cladribine combination therapy was also used to treat nine MCL patients in the study by Robak et al. (7) (2006), resulting in an OR rate of 67% and a CR rate of 22%. One report also noted a CR by colonic MCL treated with rituximab and cladribine (30). It is suggested that hypomethylating agents are one of several molecules that synergistically enhance the MCL therapy efficacy (11–14).

In this study, we demonstrated that the combination of bortezomib, cladribine, and rituximab is a well-tolerated regimen, despite the elderly study population (median age of 64 years). No severe systemic toxicity was observed during this trial. The most common AEs resulted from bone marrow suppression. The therapy was not associated with a significant rate of opportunistic infections. Antiviral and antifungal prophylaxes were not used. Only one patient receiving cladribine dose scale 3 of 5 mg/m^2^ was considered possibly experiencing DLT. A previous study that also utilized this dosing of cladribine in combination with rituximab reported one death during therapy due to cerebrovascular accident (7). This toxicity profile is much more preferable to that of more intensive R-CHOP or R-hyper-CVAD regimens.

This trial resulted in OR and CR rates of 84.6%, including OR and CR rates of 100% in the newly diagnosed subject cohort. In addition, the newly diagnosed subject cohort’s 2-year PFS rate was 90%. In the study comparing MCL patients who were treated with cladribine and rituximab versus cladribine alone, the most striking difference occurred between the duration of responses in these two groups. The 2-year PFS rate was 43% in the group that received combination therapy versus 21% in the group that received cladribine alone (7). We believe that the PFS rate is higher in this trial due to the use of an additional agent, bortezomib (31, 32). Rituximab maintenance therapy (in four cases, rituximab and bortezomib maintenance therapy) also played a role in maintaining CR in the long term (33). Our previous vorinostat (SAHA), cladribine, and rituximab (SCR) study results confirmed the importance of maintenance therapy (12).

The efficacy of the VCR combination regimen in the much smaller cohort of relapsed MCL patients in this study was not as impressive as that in the cohort of newly diagnosed MCL patients. The relapsed MCL cohort included two patients who suffered disease progression while receiving VCR treatment. Furthermore, both patients shared the features of the blastoid variant of MCL, and one of the patients had additional poor prognostic cytogenetic mutation. Our result on treating relapsed MCL patients is consistent with the results from a multicenter phase 2 PINNACLE study that utilized bortezomib to treat relapsed or refractory MCL, which resulted in an OR rate of 33% and a CR rate of 8% as well as our multicenter (8, 34, 35).

Other trials have also studied therapies for newly diagnosed MCL. Ruan et al. (36) (2015) evaluated the combination of lenalidomide plus rituximab in this patient population. Of a total of 38 patients at the median follow-up of 30 months, the OR rate among participants with newly diagnosed MCL was 92%, with a CR rate of 64% and a 2-year PFS estimated to be 85%. A separate study by Rummel et al. (37) (2013), also of newly diagnosed disease, showed that a combination of bendamustine and rituximab significantly improved PFS of elderly patients with indolent MCL compared with R-CHOP, with a median PFS of 69.5 months but a CR rate of only 40%. Prior studies of this drug combination in patients with rituximab-refractory, indolent, and transformed non-Hodgkin’s lymphoma showed a high relapse rate, with a median duration of response of 6.7 months (38).

Tremendous efforts have been made in searching for a more effective MCL treatment target (39–42). Cladribine is a promising hypomethylating agent in MCL combination therapy (1). We conducted a DNA methylation assay on two responders and two non-responders. PCA data showed that there is a difference between responders and non-responders in methylation status change after receiving VCR regimen treatment. Furthermore, the DMR methylation status change patterns between responders and non-responders in promoter regions of five genes raise the possibility of biomarkers and potential treatment targets. However, these DMR methylation data need to be verified by conducting studies in a larger patient population.

Our data suggest that the VCR combination therapy is effective in treating MCL patients with minimal toxicity. VCR should be considered a viable option for first-line therapy for elderly MCL patients or patients who opt for less intensive regimens. A phase II/III study will further confirm the efficacy of this VCR regimen and help us narrow the list of treatment-response biomarker candidates identified in this study, which also potentially could be novel treatment targets. Since dual hypomethylating agent therapy has been described in the treatment of AML (43), cladribine and bortezomib may have unique non-overlapping therapeutic effects in hematologic malignancies, especially in combination with other epigenetic agents and monoclonal antibodies/antibody–drug conjugates (1).

## Data Availability

The original contributions presented in the study are included in the article/supplementary material. Further inquiries can be directed to the corresponding authors.
